# Exploring Drug-Receptor Interaction Kinetics: Lessons from a Sigma-1 Receptor Transmembrane Biosensor

**DOI:** 10.3389/fphar.2017.00004

**Published:** 2017-01-17

**Authors:** Víctor Fernández-Dueñas, Javier Burgueño, Francisco Ciruela

**Affiliations:** ^1^Unitat de Farmacologia, Departament Patologia i Terapèutica Experimental, Facultat de Medicina, IDIBELL, Universitat de Barcelona, L'Hospitalet de LlobregatBarcelona, Spain; ^2^Institut de Neurociències, Universitat de BarcelonaBarcelona, Spain; ^3^Drug Discovery and Preclinical DevelopmentESTEVE, Barcelona, Spain

**Keywords:** σ_1_ receptor, crystal structure, cell surface, FRET, plasma membrane

An important field of study in pharmacology comprises the investigation of drug-target interaction kinetics. Thus, assessing both the lifetime of a drug on its receptor (i.e., drug-target residence time; Copeland, 2016) and the magnitude of drug-mediated receptor activation (i.e., drug efficacy) across the time are critical to understand *in vivo* pharmacological activity of small-molecule drugs. Of note, while classical *in vitro* methods view drug-receptor interaction in terms of equilibrium affinity, the residence time model considers the dynamics of receptor conformational rearrangements, which affect drug association and dissociation. Although, classical binding experiments can also address kinetics questions, they are tedious and very time consuming. Accordingly, monitoring drug-receptor interaction dynamics by means of receptor biosensors has become fundamental for understanding how drugs trigger receptor activity over the time. Precisely, in the last years, a number of Fluorescence Resonance Energy Transfer (FRET)-based assays have been developed to accurately display drug-receptor interaction in real time (Lohse et al., 2012). Indeed, one of the most outstanding methods consists of assessing intramolecular conformational rearrangements upon receptor challenge by monitoring intramolecular FRET changes (Vilardaga et al., 2009). Thus, a FRET-based receptor biosensor is built by fusing both donor and acceptor fluorophores to the receptor sequence (Vilardaga et al., 2009). Importantly, a general consensus has prompted to basically attach these molecules (i.e., cyan and yellow fluorescent proteins, CFP and YFP, respectively) intracellularly, this is, in the cytosolic side of the receptor's structure (Figure 1A). Accordingly, when the receptor is activated and a conformational rearrangement occurs the distance and/or orientation of the fluorophores within the receptor biosensor changes and it is possible to monitor FRET changes in real time, thus permitting to finely characterize receptor's activation. Needless to say, although precision is higher than that obtained in classical binding assays, the present biosensors cannot discern between receptors expressed at the cell surface or intracellularly, thus much effort is needed in order to exactly elucidate ligand-receptor kinetics constants.

**Figure 1:**
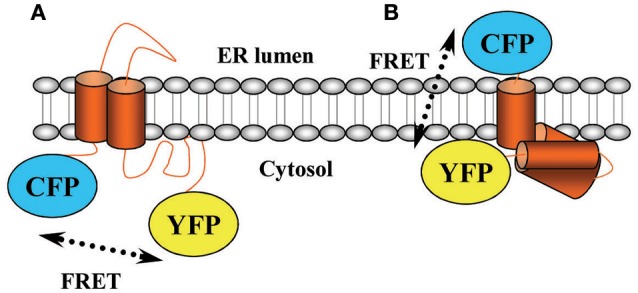
**Scheme of the σ1R biosensor**. A schematic diagram illustrating the topology of the σ1R as considered before **(A)** and after **(B)** its crystallization. ER, endoplasmic reticulum.

Within this scenario, we recently developed a dynamic σ1 receptor (σ1R) biosensor able to achieve ligand-specific conformational rearrangements with high temporal resolution (Gómez-Soler et al., 2014). The σ1R gene encodes an endoplasmic reticulum and plasma membrane anchored protein of 223 amino acids, which shows no similarity to any other known mammalian protein receptor but that is quite conserved across species (Vela et al., 2015). The σ1R has been lately defined as a ligand-regulated chaperone that is able to interact with a myriad of other proteins including receptors, enzymes or ion channels. For instance, it has been linked to serotonin or NMDA receptors, which may be of interest for the management of neurological or neurodegenerative disorders (Nguyen et al., 2015). In fact, this latter topic has recently gained a great interest, thus a number of animal models have been developed to study neuroprotective effects of σ1R ligands (Nguyen et al., 2015). Needless to say, a σ1R-defficient mouse model was developed, which exhibited no overt phenotype in terms of baseline behavior or immune profile (Langa et al., 2003). Hence, the σ1R may basically modulate the intracellular signaling evoked by those interacting partners rather than by exerting an action by itself (Romero et al., 2012). Due to the lack of this direct functional effect or signaling by its own, it has been difficult to classify σ1R ligands by their intrinsic activity and time-consuming *in vivo* tests are frequently used to determine the functional characteristics of σ1R ligands. Accordingly, we designed a CFP/YFP-tagged σ1R aiming to distinguish agonists from antagonists based on the conformational rearrangement engaged. We thus attached CFP and YFP to the N- and C-terminus of the receptor based on the most accepted structural model available, in which both extremes of the receptor faced the cytoplasm (Ruoho et al., 2012). Of note, a few months ago, the crystal structure of the human σ1R was finally disclosed (Schmidt et al., 2016). This seminal study revealed that the σ1R holds a triangular structure comprising three tightly associated protomers, each with a single transmembrane domain that segregates the N- and C-terminus of the receptor (Schmidt et al., 2016) (Figure 1B). Indeed, previous biochemical studies also supported the contention that σ1R do oligomerize (Gromek et al., 2014; Mishra et al., 2015). Interestingly, the σ1R oligomerization status seems to be ligand-dependent, thus while antagonists stabilize high molecular mass oligomers the agonists prompt the dissociation of these complexes (Mishra et al., 2015). Collectively, these results suggest that σ1R oligomerization might play a key role in receptor functioning by controlling ligand efficacy (Schmidt et al., 2016).

In view of the σ1R molecular structure, we blindly designed a transmembrane biosensor, since we thought that both CFP and YFP were inside the cell while they were really in opposite sides of the cell surface (Figure 1B). Nevertheless, we observed by means of donor recovery after acceptor photo bleaching that there was constitutive intramolecular FRET (Gómez-Soler et al., 2014). And subsequently, once ascertained that the biosensor retained its essential ligand binding properties we performed real-time FRET, in which the superfusion of σ1R agonists or antagonists led to a decrease or increase, respectively, of energy transfer (Gómez-Soler et al., 2014). Since it has been postulated that σ1R antagonists are analgesic and agonists preclude opioid-mediated pain relief (Prezzavento et al., 2010), we assessed antinociception elicited by σ1R ligands in the formalin pain animal model to correlate with the FRET-induced changes. Hence, while σ1R antagonists led to a FRET increase and produced analgesia, agonists led to a FRET decrease and did not induce antinociceptive effects (Gómez-Soler et al., 2014).

The development of our σ1R biosensor permitted to classify σ1R ligands according to its intrinsic activity, since it was possible to correlate FRET changes upon ligand activation with analgesic efficacy. This tool may therefore represent a powerful approach in drug discovery in the intriguing world of the σ1R. But on the other hand, it may also represent the beginning of a new generation of biosensors able to characterize ligand-receptor kinetics constants with high precision. In such way, as commented above, we blindly developed the first receptor biosensor able to detect intramolecular FRET changes through the cell surface. Needless to say, although scarcely assayed, transmembrane FRET has been previously described; thus, it was demonstrated that energy transfer is possible between fluorophores despite they are segregated by a lipid bilayer (Majoul et al., 2001; Haga et al., 2012). Taken together, data support that conformational receptor rearrangement can be monitored by means of FRET across the plasma membrane. Thus, in order to exclusively target receptors expressed at the cell surface different approaches would be intended. For instance, by substituting the outer-fluorophore (i.e., CFP) by another protein (i.e., O-6-methylguanine-DNA methyltransferase, AGT), which may be selectively labeled when expressed at the plasma membrane (Maurel et al., 2008). Upon these conditions, when challenging the new biosensor with selective ligands, the FRET process would only occur with these receptors expressed at the cell surface, thus permitting to perform the most real-time precise kinetics.

Overall, pharmacologists have explored for a long time the activity of σ1R based on structural models that have been finally overruled by the recent elucidation of its structure (Schmidt et al., 2016). The precise knowledge of σ1R structure may not only prompt to review a number of previous inaccurate conclusions with direct impact on the σ1R pharmacology, but it also may lead to design novel strategies to wholly characterize ligand-receptor interaction kinetics. In conclusion, the delayed discovery of the first transmembrane biosensor may represent a kind of paradigm shift. Hence, after witnessing an apple falling from the tree, a new frontier has been torn down to develop novel tools for studying receptors' pharmacology.

## Author contributions

VF wrote the paper. JB wrote the paper. FC conceived the idea and wrote the paper.

### Conflict of interest statement

The authors declare that the research was conducted in the absence of any commercial or financial relationships that could be construed as a potential conflict of interest.
